# Are Drugs Associated with Microscopic Colitis? A Systematic Review and Meta-Analysis

**DOI:** 10.3390/diseases11010006

**Published:** 2022-12-29

**Authors:** Zahid Ijaz Tarar, Umer Farooq, Mustafa Gandhi, Faisal Kamal, Moosa Feroze Tarar, Veysel Tahan, Harleen Kaur Chela, Ebubekir Daglilar

**Affiliations:** 1 Department of Medicine, University of Missouri, Columbia, MO 65212, USA; 2 Department of Medicine, Rochester General Hospital, Rochester, NY 14621, USA; 3 Division of Gastroenterology, University of California, San Francisco, CA 94143, USA; 4 Department of Medicine, Services Institute of Medical Sciences, Lahore 54000, Pakistan; 5 Division of Gastroenterology and Hepatology, University of Missouri, Columbia, MO 65212, USA; 6 Division of Gastroenterology and Hepatology, Charleston Area Medical Center, West Virginia University School of Medicine, Charleston, WV 25303, USA

**Keywords:** microscopic colitis, drug use, PPIs, NSAIDs, SSRIs, statins

## Abstract

There is growing evidence of the association of Microscopic Colitis (MC) with the use of specific medications such as proton pump inhibitors (PPIs), Selective serotonin reuptake inhibitors (SSRIs), Non-Steroidal anti-inflammatory drugs (NSAIDs), Statins and H2-receptor antagonists (H2RA). In our study, we calculated the pooled odds of MC in patients using these drugs. We performed a detailed search of major databases, including PubMed/Medline, Scopus, web of science, and Embase, to include the studies in which odds of MC were reported after using above mentioned drugs. A random-effects model was used to pool the estimates. Thirteen studies were included in our analysis consisting of 304,482 patients (34,194 cases and 270,018 controls). In eight studies, the control group consisted of a random population selected based on age, gender and same birth year, whereas 3 studies recruited patients who presented with diarrhea and underwent colonoscopy and biopsy to rule out MC. Two studies reported odds of MC for both diarrhea and random control groups. Patients taking PPIs were more likely to develop MC, AOR 2.65 (95% CI 1.81–3.50, *I*^2^ 98.13%). Similarly, higher odds of association were found in patients taking SSRIs (OR 2.12, 95% CI 1.27–2.96, *I*^2^ 96.46%), NSAIDs (OR 2.02, 95% CI 1.33–2.70, *I*^2^ 92.70%) and Statins (OR 1.74, 95% CI 1.19–2.30, *I*^2^ 96.36%). No difference in odds of developing MC was seen in patients using H2RA compared to the control group (OR 2.70, 95% CI 0.32–5.08, *I*^2^ 98.67%). We performed a subgroup analysis based on the control group and found higher odds of MC in patients on PPIs compared to the random control group (OR 4.55, 95% CI 2.90–6.19, *I*^2^ 98.13%). Similarly, higher odds of MC were noted for SSRI (OR 3.23, 95% CI 1.54–4.92, *I*^2^ 98.31%), NSAIDs (OR 3.27, 95% CI 2.06–4.48, *I*^2^ 95.38%), and Statins (OR 2.23, 95% CI 1.41–3.06, *I*^2^ 98.11%) compared to the random control group. Contrary lower odds of MC were seen in the PPI and H2RA group compared to the diarrhea control group (OR 0.68, 95% CI 0.48–0.88, *I*^2^ 7.26%), (OR 0.46, 95% CI 0.14–0.78, *I*^2^ 0%) respectively. We found no difference in odds of MC in patients on SSRIs (OR 0.96, 95% CI 0.49–1.42, *I*^2^ 37.89%), NSAIDs (OR 1.13, 95% CI 0.49–1.76, *I*^2^ 59.37%) Statins (OR 0.91, 95% 0.66–1.17, *I*^2^ 0%) and H2RA (OR 3.48, 95% CI −0.41–7.36, *I*^2^ 98.89%) compared to the diarrhea control group. We also analyzed the association use of PPIs and NSAIDs with the development of collagenous colitis (CC) and lymphocytic colitis. Only the use of NSAIDs was associated with increased odds of developing collagenous colitis (OR 1.61, 95% CI 1.50–1.72, *I*^2^ 0%). No increased odds of CC and LC were seen in PPI users. PPIs, NSAIDs, SSRIs, and Statins are associated with an increased risk of MC compared to the random control group. On the contrary, the use of PPIs, NSAIDs, SSRIs, and Statins is not associated with an increased risk of MC when compared to the diarrhea control group.

## 1. Introduction

Microscopic colitis (MC) is a chronic inflammatory disease of the large intestine, consistent with two histological subtypes, lymphocytic colitis (LC) and collagenous colitis (CC). The most common presentation of MC is chronic watery diarrhea associated with abdominal pain, fecal urgency, and incontinence [1,2]. Microscopic colitis was considered a disease of old age, but now the incidence is rising in the younger population. The updated incidence and prevalence of MC are 25.8 cases/per 100,000 and 246.2/per 100,000, as reported by Tome et al. in an epidemiological study performed in Olmsted County, MN, USA [3,4]. The possible explanation for the increasing incidence of MC is better awareness and understanding of the disease and better and readily available diagnostic modalities such as endoscopy and biopsy [5].

The inflammation of the colon in response to luminal antigen exposure is suggested to underlie the mechanism of MC, but the exact pathogenesis is still unclear [6]. Endoscopic examination in MC usually reveals normal mucosa. Diagnosis is often established with a microscopic examination, which shows increased intraepithelial lymphocytes, inflamed lamina propria, and damage to the epithelial surface in both LC and CC [7,8]. The histological presence of collagenous bands allows for the differentiation between the two subtypes of MC and is only seen in CC [1,9].

Female sex and increasing age are the established risk factors associated with MC [3]. There is growing evidence that MC is related to other autoimmune diseases such as celiac disease, thyroid disorders, and rheumatic diseases, and the use of certain medications such as proton pump inhibitors (PPIs), Selective serotonin reuptake inhibitors (SSRIs), Non-steroidal anti-inflammatory drugs (NSAIDs), and Statins [2,10,11,12,13]. 

In our meta-analysis, we aimed to study the association of PPIs, SSRIs, NSAIDs, Statins, and H2RA with microscopic colitis. We further examined the association of PPIs and NSAIDs with subtypes of MC, including subgroup analysis for LC and CC. This is the first systematic review and meta-analysis on this topic to the best of our knowledge.

## 2. Methods

### 2.1. Data Search and Screening

We designed and performed an electronic literature search of Medline/PubMed, Embase Ovid, Cochrane Central Register for controlled trials, Scopus, and web of science from inception to 30 September 2022. We followed the preferred reporting items for the systematic review and meta-analysis (PRISMA) statement. Zahid Tarar (ZT) and Umer Farooq (UF) designed the search strategy, which was approved by Ebubekir Daglilar (ED). Both ZT and UF independently searched the databases mentioned above and registers. We designed three questions for our analysis. (1) Is there an association between medication use and microscopic colitis? (2) what are the odds of developing microscopic colitis in patients taking certain medications, including PPIs, SSRIs, NSAIDs, Statins, and H2RA? (3) what are the odds of developing collagenous and lymphocytic colitis in patients taking PPIs and NSAIDs? We included medical subject headings [Mesh] and free-text terms in our search. Following free text terms were used in different combinations: “(Microscopic colitis) AND (Drugs) OR (Medications) AND (Proton pump inhibitors) AND (PPIs OR SSRIs OR Statins OR H2RA OR NSAIDs) AND (Collagenous Colitis) AND/OR (Lymphocytic Colitis)”. All search fields were used in all databases and registers except in Scopus, where the “Article title, keywords, and abstract” field was used. We also hand-searched the reference list of included studies.

### 2.2. Eligibility Criteria and Study Selection

We included the studies which meet the following criteria. (1) Reported odds of microscopic colitis in patients taking PPIs, SSRIs, NSAIDs, Statins, or H2RA; (2) Defined a control group; (3) Age above 18; (4): Reported odds of developing LC and CC while on PPIs or NSAIDs. 

The following exclusion criteria were used (1) Studies in which odds ratios were not provided, or just *p*-value was provided; (2) Case reports or case series; (3) Abstracts or conference articles; (4) Letter to editors, Review articles and editorials; (5) Studies in which outcome data were missing; (6) Studies in a foreign language. Two investigators (ZT and UF) independently screened the abstracts, titles, and complete reports to identify the eligible studies based on pre-defined inclusion criteria. Any conflict or disagreement between the reviewers was resolved through discussion or by a third reviewer (MG). All references were downloaded in Endnote 11, and duplicate studies were removed manually and automatically.

### 2.3. Data Extraction

Data were extracted into Microsoft excel by two reviewers (ZT and MT). They independently pulled the information about study design, first author, year of publication, country of study, study cohort characteristics (age, sex, sample size), duration of the study, period of medication use, percentage of patients taking medications in both cases and control group, odds of developing MC in patients taking drugs, odds of developing CC and LC in patients taking PPIs or NSAIDs. We did not conduct an analysis on the odds of LC and CC for stains, SSRIs, and H2RA due to a lack of data availability. Once data was extracted, a third reviewer (ED) independently reviewed the extracted data sheet, and the final data sheet was prepared after discussion.

### 2.4. Statistical Analysis

We calculated the pooled odds ratio from each study’s reported adjusted odds ratios. A random-effects model was used to calculate the pooled Odds ratio with 95% CI, and *p*-value < 0.05 was considered statistically significant. Cochrane chi-square test and *I*^2^ statistics were used to test heterogeneity. Heterogeneity of 0, 25%, 50% and 75% were interpreted as absent, low, moderate and high, as described by the Cochrane Handbook for Systematic review [14]. Forest plots were created to report the results. The funnel plot and nonparametric trim and fill analysis for asymmetry were used to assess the publication bias. We used STATA software 17 to conduct the meta-analysis.

## 3. Results

### 3.1. Search Results and Study Characteristics

**Figure 1:**
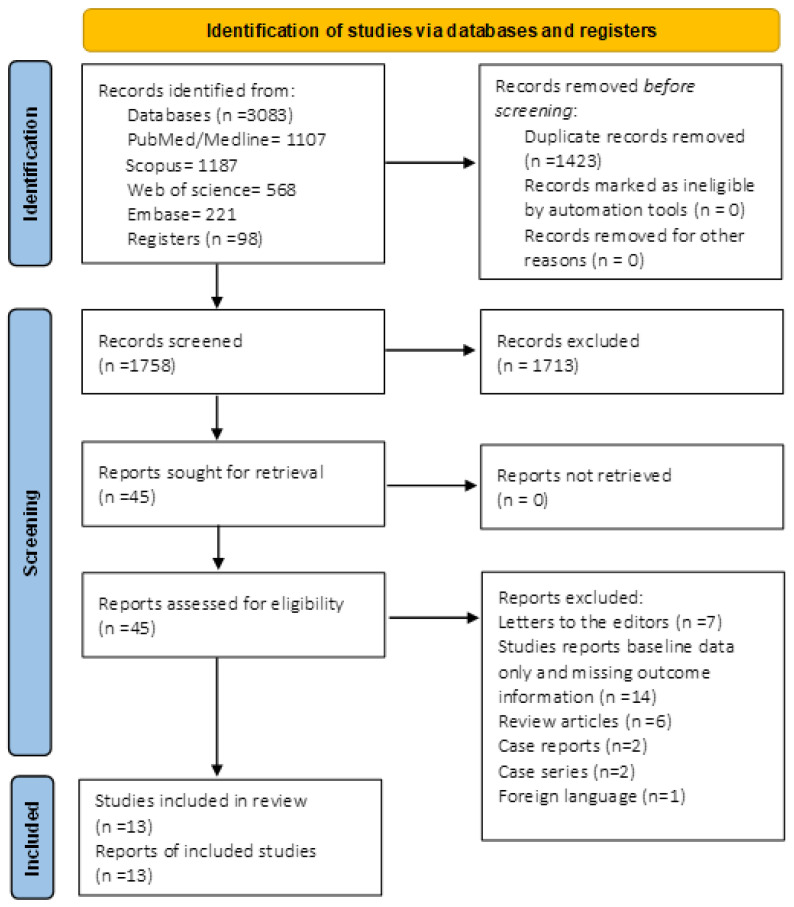
Prisma flow diagram for search and selection process of meta-analysis.

Figure 1 outlines the summary of the selection process. On the initial search of electronic databases and registers, we identified 3181 reports (1107 from PubMed/Medline, 1187 from Scopus, 568 from web of science, 221 from Embase, and 98 from Registers). After removing 1423 duplicate records, 1758 articles were screened, and finally, 45 reports were considered for eligibility. Of these 45 articles, 13 studies fulfilled the selection criteria and were included in the final analysis. Eight studies [11,13,15,16,17,18,19,20] used random control adjusted for age, sex, GP practice, or birth year.

In contrast, three studies [8,9,12] used diarrhea controls in which colonoscopy and biopsy were performed to rule out microscopic colitis. Masclee et al. [21] and Pascua et al. [22] reported the odds of MC in both random community controls and diarrhea controls. In five studies [9,11,15,16,20], odds of collagenous and lymphocytic colitis were calculated in patients on either PPIs or NSAIDs. Supplementary Table S1 details the characteristics of included studies.

### 3.2. Pooled Odds of MC in Patients Taking PPIs and Subgroup Analysis Based on Control Groups

**Figure 2:**
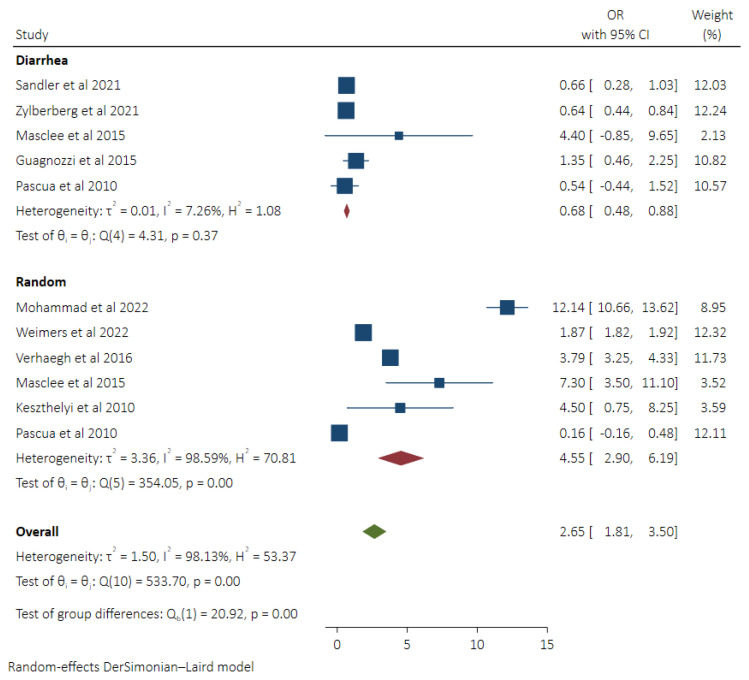
Pooled odds of MC in PPI users with subgroup analysis based on control groups (Diarrhea vs. Random Control) [8,9,12,13,17,18,19,21,22].

Nine studies [8,9,12,13,17,18,19,21,22] reported the odds of microscopic colitis in patients using proton pump inhibitors. Studies by Masclee et al. [21] and Pascua et al. [22] reported two different Odds ratios based on the control groups (Community random and diarrhea controls). Pooled Odds of MC in patients taking PPIs were 2.65 (95% CI 1.81–3.50, *I*^2^ 98.13%). On subgroup analysis, lower odds of MC (OR 0.68, 95% CI 0.48–0.88, *I*^2^ 7.26%) were found compared to the diarrhea control group, while greater odds were seen compared to the random control group (OR 4.55, 95% CI 2.90–6.19, *I*^2^ 98.13%). A statistically significant difference was seen between the groups (*p* < 0.0001) (Figure 2).

### 3.3. Pooled Odds of MC in Patients Taking SSRIs and Subgroup Analysis Based on Control Groups

**Figure 3:**
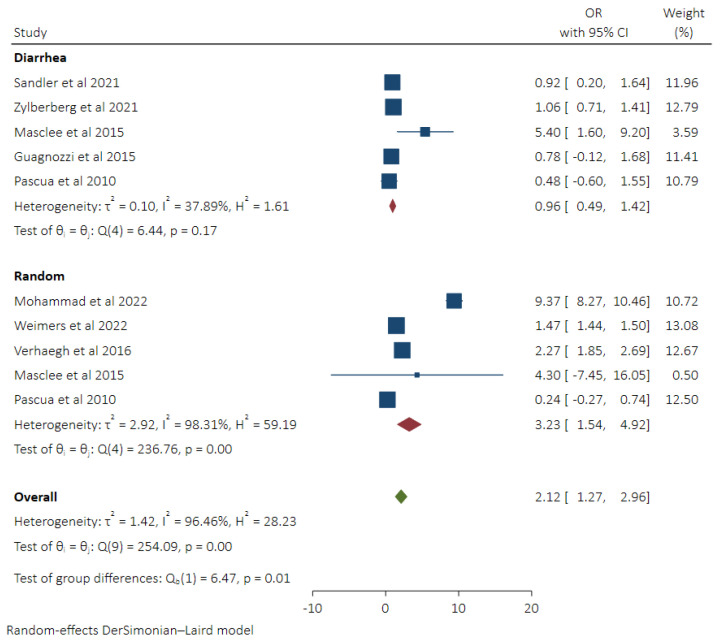
Pooled odds of MC in SSRI users with subgroup analysis based on control groups (Diarrhea vs. Random Control) [8,9,12,17,18,19,21,22].

Higher pooled odds of MC were observed in patients taking SSRI (OR 2.12(95% CI 1.27–2.96, *I*^2^ 96.46%). We included nine different odds ratios from 8 studies [8,9,12,17,18,19,21,22] to calculate the pooled odds ratio because Pascua et al. [22] provided two ratios based on two different control groups. Greater odds of MC were seen in patients taking SSRIs when compared to random health controls (OR 3.23, 95% CI 1.54–4.92, *I*^2^ 98.31%), whereas on the contrary, no difference in odds of MC was noted in comparison to the diarrhea control group (OR 0.96, 95% CI 0.49–1.42, *I*^2^ 37.89%) (Figure 3).

### 3.4. Pooled Odds of MC in Patients Taking NSAIDs and Subgroup Analysis Based on Control Groups

**Figure 4:**
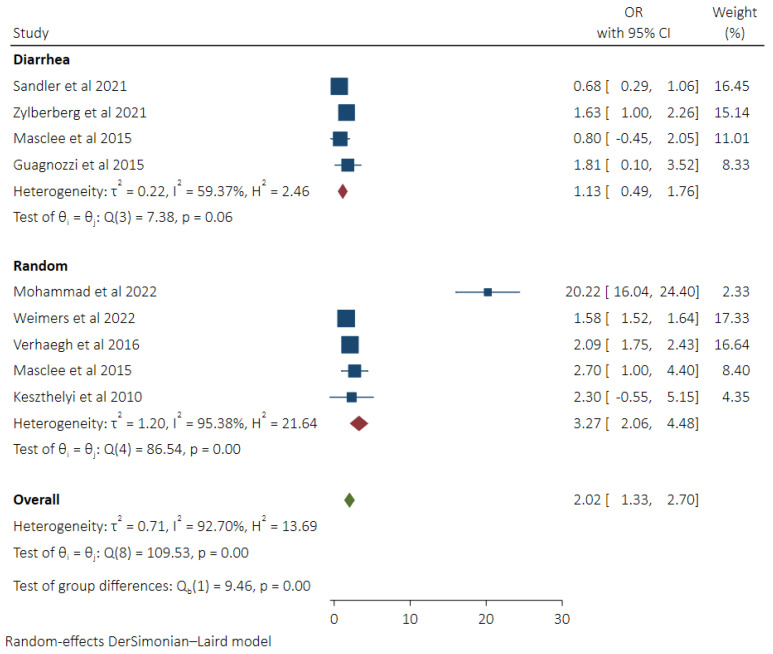
Pooled odds of MC in NSAIDs users with subgroup analysis based on control groups (Diarrhea vs. Random Control) [8,9,12,13,17,18,19,21].

Eight studies [8,9,12,13,17,18,19,21] provided the adjusted odds of MC in patients who were currently taking NSAIDs or were on them in the past. Patients taking NSAIDs had greater odds of developing MC (OR 2.02, 95% CI 1.33–2.70, *I*^2^ 92.70%). On subgroup analysis based on the control group, we found higher odds of MC in patients taking NSAIDs in comparison to the random control group (OR 3.27, 95% CI 2.06–4.48, *I*^2^ 95.38%), whereas no difference in odds of MC was noted when compared to the diarrhea control patient group (OR 1.13, 95% CI 0.49–1.76, *I*^2^ 59.37%). A statistically significant difference was noted between these two groups p (0.001) (Figure 4).

### 3.5. Pooled Odds of MC in Patients Taking Statins with Subgroup Analysis of Diarrhea versus Random Controls

**Figure 5:**
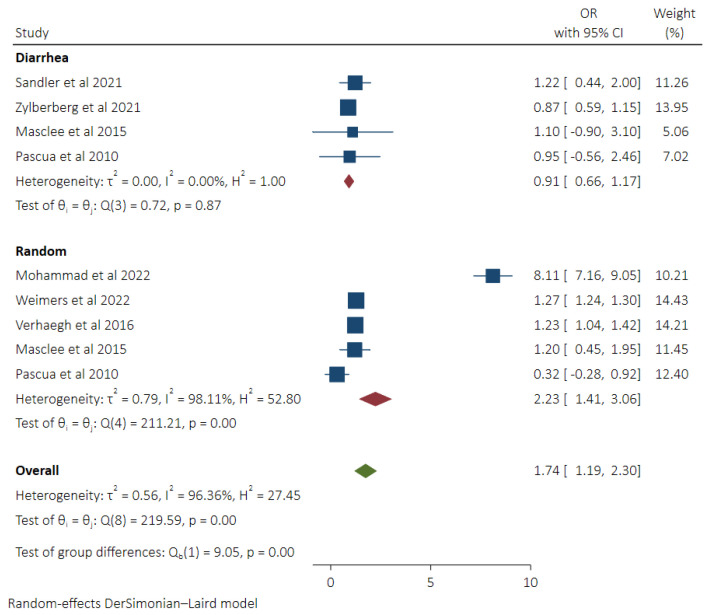
Pooled odds of MC in Statin users with subgroup analysis based on control groups (Diarrhea vs. Random Control) [8,9,17,18,19,21,22].

Pooled odds of MC in statin users were 1.74 (95% CI 1.19–2.30, *I*^2^ 96.36%) calculated from 9 adjusted odd ratios obtained from 7 studies [8,9,17,18,19,21,22]. Higher odds of MC were seen in patients taking statins compared to the healthy random control group (OR 2.23, 95% CI 1.41–3.06, *I*^2^ 98.11%). No significant difference in odds of MC was found in statin users compared to the diarrhea control group (OR 0.91, 95% CI 0.66–1.17, *I*^2^ 0%) (Figure 5).

### 3.6. Pooled Odds of MC in Patients Taking H2RA and Subgroup Analysis Based on Control Groups

**Figure 6:**
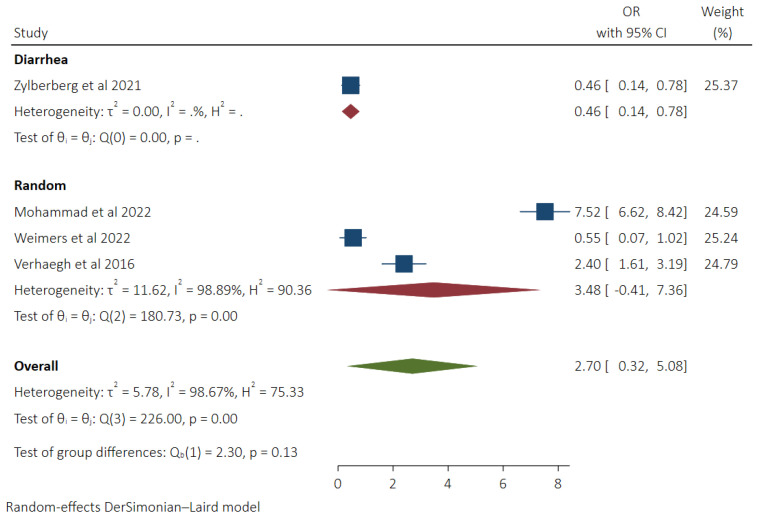
Pooled odds of MC in H2RA users with subgroup analysis based on control groups (Diarrhea vs. Random Control) [9,17,18,19].

H2 receptor antagonist use was not associated with increased odds of MC (OR 2.70, 95% CI −0.32–5.08, *I*^2^ 98.67%) based on the data provided in four studies [9,17,18,19]. Lower odds of MC were seen in patients using H2RA compared to the diarrhea control group (OR 0.46, 95% CI 0.14–0.78, *I*^2^ 0%). Similar odds of MC were seen compared to the community control patient population (OR 3.48, 95% CI −0.41–7.36, *I*^2^ 98.89%). No significant difference was seen between the groups (*p* 0.13) (Figure 6).

### 3.7. Subgroup Analysis on the Association of LC and CC with the Use of PPIs and NSAIDs

**Figure 7:**
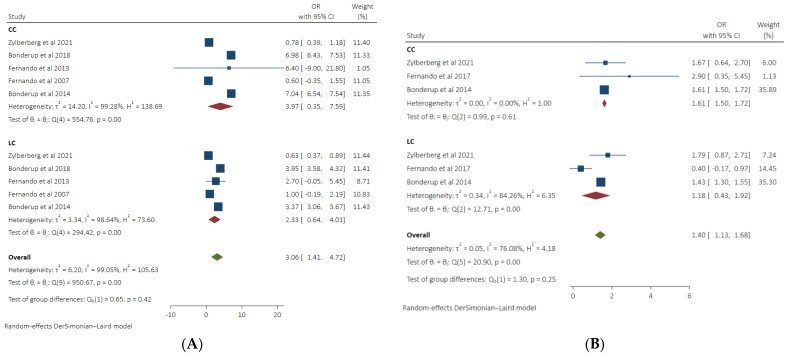
(**A**): Pooled odds of Lymphocytic and collagenous colitis in PPI users. (**B**): Pooled odds of Lymphocytic and collagenous colitis in NSAIDs users [8,9,12,13,17,18,19,21,22].

We performed a subgroup analysis to determine the risk of developing collagenous colitis or lymphocytic colitis in patients taking proton pump inhibitors or NSAIDs. Similar odds of CC and LC (3.97, 95% CI 0.35–7.59, *I*^2^ 99.28%), (2.33, 95% CI 0.64–4.01, *I*^2^ 98.64%) respectively were seen in patients taking PPIs compared to control group. No statistically significant difference was seen between both groups (*p* 0.42). Odds of CC (OR 1.61, 95% CI 1.50–1.72, *I*^2^ 0%) were significantly higher in patients on NSAIDs, whereas similar odds of LC were found in NSAIDs users (OR 1.18, 95% CI 0.43–1.92, *I*^2^ 84.26%) compared to the control group. No significant difference between the CC and LC groups was seen (*p* 0.25) (Figure 7A,B).

## 4. Quality Assessment

We evaluated the quality of included studies using the Methodological Index for Nonrandomized Studies (MINORS) criteria [23]. MINORS criteria score such studies on twelve items. Individual items are scored from 0 to 2 (2 when reported and adequate; 1 when inadequately reported; 0 if not reported). Scores from each item were summed up. The quality of studies was classified as high quality (≥11), fair (score 6–10), or poor (score ≤ 5). Two authors (UF and ZT) conducted the quality assessment separately, and any disagreement was resolved by consensus with a third reviewer (FK). The quality assessment of studies is summarized in Supplementary Table S2.

## 5. Publication Bias

Visible asymmetry was noted on funnel plots, but the nonparametric trim and fill test was negative for any publication bias (Supplementary Figure S1a–e).

## 6. Discussion

In our analysis, we reported the effect of medication use on the development of microscopic colitis. We demonstrated that certain medications such as proton pump inhibitors, SSRIs, NSAIDs, and Statins are associated with an increased risk of MC, but H2RA use was not associated with an increased risk of MC when compared to random control groups. This is the first meta-analysis on this topic to the best of our knowledge, and the results of our analysis are significant.

Our results showed that using PPIs is associated with significantly higher odds of MC, in accordance with the results of the previous studies [12,18,19,21]. It is postulated that changes in gut flora, electrolyte imbalance due to acid suppression, and intestinal dysbiosis caused by PPIs are the possible underlying mechanism of MC development [24,25,26]. The noteworthy result in our analysis is that when MC cases were compared with diarrhea controls, the use of PPI was associated with decreased pooled odds of MC, while in studies in which random controls were selected, the risk of MC in PPI users was high. These results raise a question about the association of MC with PPI use because patients who suffer from gastrointestinal symptoms are more likely to get a PPI prescription compared to the healthy control group, and a similar observation has been made by Law et al. [27]. On subgroup analysis, it was found that the use of PPI was not associated with a greater likelihood of CC or LC. Lower odds of MC compared to diarrhea control and higher odds with random control warrant further prospective trials to establish or negate the association of MC with PPIs.

Data on the association of MC with the use of H2RA is conflicting. As the effect of H2RA on acid secretion is like PPIs, it is thought that they can cause intestinal dysbiosis, leading to MC. Our analysis did not find increased odds of MC in patients taking H2RA. A recent study by Zylberberg et al. [9] reported lower odds of MC in patients taking H2RA. In another study by Mohammad et al. [17], significantly higher odds of MC in association with H2RA were reported. The noteworthy fact is that Zylberberg et al. [9] recruited a diarrhea control group as a comparison, while Mohammad et al. [17] conducted a population-based study.

The NSAIDs inhibit prostaglandin synthesis, which results in increased gut permeability and impairs the integrity of the mucosal barrier resulting in an influx of bacteria and toxins into the intestinal lumen. The reaction to these luminal antigens is considered an underlying pathogenetic factor for the development of MC in NSAID users [17,28]. We reported significantly higher odds of MC in patients taking NSAIDs, and these results reinforce the results of previously conducted studies [9,12,13,17,19,21,29]. We also found that the odds of developing CC are higher in NSAID users, though no significantly higher risk of LC was seen in patients taking NSAIDs. On subgroup analysis based on the control group, we demonstrated that when MC cases using NSAIDs were compared to the diarrhea control group, no difference in risk of MC was seen, while when MC cases were compared to a healthy random control group, significantly greater odds of MC were noted. This discrepancy in results based on the control group in PPI and NSAIDs group warrants us to be careful about interpreting these results.

We demonstrated greater odds of MC in statin users, which is consistent with the results reported in the studies performed earlier [8,17,18,19]. In subgroup comparison to diarrhea and random control groups, no difference in odds of MC was seen. The underlying mechanism involved in MC development in statin users is not precise; however, inflammatory effects resulting from the downregulation of anti-tumor necrosis factor and upregulation of pro-inflammatory cytokines such as IL-8 by statin can contribute [17,30,31].

The effect of SSRI on the causation of MC is not well studied, although a few factors which have been considered potential contributors and reported in the literature are the following. First, it is said that serotonin possesses inflammatory characteristics, as seen in cases of colitis [32]. Moreover, a higher number of enterochromaffin cells are seen in MC patients, which secrete more serotonin. This results in the upregulation of immune-mediated markers by activating the nervous system, enhancing chloride secretion and gut motility [33,34]. We demonstrated that patients on SSRI are associated with greater odds of developing MC, which is consistent with the finding in most of the studies we included in our analysis. We did not find any difference in the odds of MC compared to diarrhea or random healthy controls.

Our study has several strengths. We conducted a detailed literature search of all the major database engines and a manual search of the bibliography of included studies. Two investigators searched and screened the databases separately, and a third reviewer approved the final studies included in the analysis. We emailed the primary and corresponding authors to get any missing information and avoid duplication of results. We performed a subgroup analysis based on control groups to prevent overestimating the effects from reports where healthy controls were recruited. We used a random-effect model to provide a more conservative and generalized pooled odds of MC in patients on different medications. We also did a sensitivity analysis to minimize the effect of any study affecting the results. Moreover, this is the first meta-study analyzing the association of MC with a particular medication.

There are a few limitations of our meta-analysis. The first few studies used random healthy controls as a comparison. In contrast, in other studies, diarrhea controls were recruited, leading to an overestimation of results due to differences in medication exposure between both control groups. Second, drug dosage was not provided in most of the included reports. Furthermore, it was not clearly stated if a patient was taking two medications which can result in MC at the time of the study. Moreover, only a few studies separately provided LC and CC odds. In addition, many reported medications are available over the counter in many countries where these studies are performed, so noted exposure can be overestimated or even underestimated.

## Data Availability

This analysis is performed on publicly available data, which can be shared on request.

## Supplementary Materials

The following supporting information can be downloaded at: https://www.mdpi.com/article/10.3390/diseases11010006/s1.
