# Supplementary material for: Are Drugs Associated with Microscopic Colitis? A Systematic Review and Meta-Analysis
Source: Diseases. 2022 Dec 29;11(1):6. doi: 10.3390/diseases11010006 (PMC9844498; doi:10.3390/diseases11010006)
Supplement: Supplementary file 1 [file diseases-11-00006-s001.zip › Figure S1. Figure Legends MC meta.pdf]

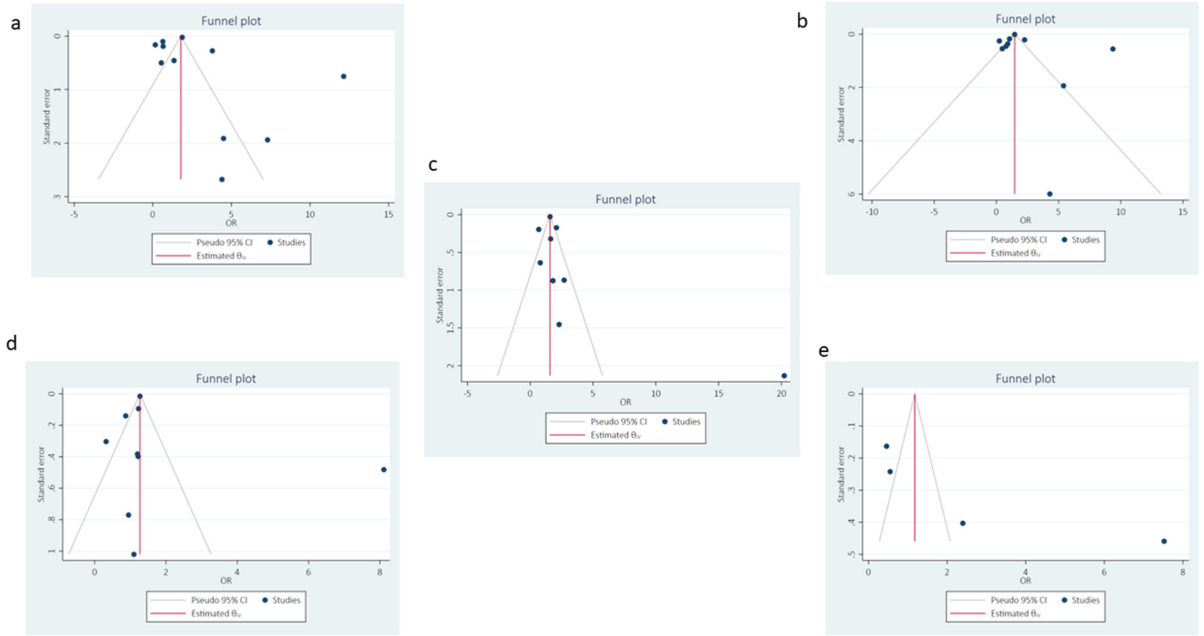


Supplementary figure S1: (a) Funnel plot for publication bias in studies assessing MC in PPI users. (b) Funnel plot for publication bias in studies assessing MC in SSRI users. (c) Funnel plot for publication bias in studies assessing MC in NSAID users. (d) Funnel plot for publication bias in studies assessing MC in Statin users. (e): Funnel plot for publication bias in studies assessing MC in H2RA users
