# Supplementary material for: Are Drugs Associated with Microscopic Colitis? A Systematic Review and Meta-Analysis
Source: Diseases. 2022 Dec 29;11(1):6. doi: 10.3390/diseases11010006 (PMC9844498; doi:10.3390/diseases11010006)
Supplement: Supplementary file 1 [file diseases-11-00006-s001.zip › Tabld S2. Quality assesment- Minors MC meta.pdf]

Study	Clearly stated aim	Inclusion of consecutive patients	Prospective collection of data	Endpoints appropriate to the aim of the study	Unbiased assessment of the study endpoint	Follow-up period appropriate to the aim of the study	Loss to follow up less than 5%	Prospective calculation of the study size	An adequate control group	Contemporary groups	Baseline equivalence of groups	Adequate statistical analyses	Total Score
Bonderup et al.2018	2	2	2	2	0	2	2	0	2	2	0	2	18
Fernandez-Banares et al.	2	2	2	2	0	2	2	0	2	2	0	2	18
Fernandez-Banares et al.	2	2	2	2	0	2	2	0	2	2	1	2	19
Guagnozzi et al.	2	2	2	2	0	2	2	0	2	2	1	2	19
Keszthelyi et al.	2	2	2	2	0	2	2	0	2	2	0	2	20
Masclee et al.	2	2	2	2	0	2	2	0	2	2	1	2	19
Mohammed et al.	2	2	2	2	0	2	2	0	2	2	0	1	17
Pascua et al.	2	2	2	2	0	2	2	0	2	2	1	2	19
Sandler et al.	2	2	2	2	0	2	0	0	2	2	1	2	17

Verhaegh et al.	2	2	2	2	0	2	2	0	2	2	1	2	19
Weimers et al.	2	2	2	2	0	2	2	0	2	2	1	2	19
Zylberberg et al.	2	2	2	2	0	2	2	0	2	2	1	2	19
Bonder Up et al 2014	2	2	2	2	0	2	2	0	2	2	0	2	18

Supplementary Table S2: Quality assessment of included studies using Minors criteria.
