# Supplementary material for: Are Drugs Associated with Microscopic Colitis? A Systematic Review and Meta-Analysis
Source: Diseases. 2022 Dec 29;11(1):6. doi: 10.3390/diseases11010006 (PMC9844498; doi:10.3390/diseases11010006)
Supplement: Supplementary file 1 [file diseases-11-00006-s001.zip › Table S1. Study Table.pdf]

| Study name | Study design and study period | Age cases | Age control | Total N | Male | Female | cases | Control |
|---|---|---|---|---|---|---|---|---|
| Mohammad et al. 2022 | Retrospective Cohort (1999-2018) | NA | NA | 1130 | 880 | 250 | 1130 | Number not specified |
| Sandler et al 2021 | Case control (2015-2020) | 63.2±12.7 | 54.5±11 | 362 | 92 | 270 | 110 | 252 |
| Weimer et al, 2022 | Case-control (2001-2016) | 65 | 67 | 156,783 | 4175 | 10127 | 14302 | 142481 |
| Zylberberg et al 2021 | Case control (2007-2016) | 63.1±15.6 | 63.1±15.7 | 1012 | 289 | 723 | 344 | 668 |
| Bonderup et al, 2018 | Case-control 2004-2013 | 68(59-77) | 68(59-77) | 112,303 | 32,714 | 79,589 | 10,652 | 101,381 |
| Verhaegh et al, 2016 | Case Control (1992-2013) | 63.3 | 63.2 | 7252 | 1943 | 5309 | 1211 | 6041 |
| Masclee et al, 2015 | Case-control (1991-2012) | 45.3±2 | 45.7±1.7 | 15,738 | 4056 | 11682 | 218 | 15520 |
| Guagnozzi et al, 2015 | Case control (2008-2011) | 50.2±20.2 | 48.6±17.8 | 363 | 142 | 221 | 46 | 317 |
| Keszthelyi et al, 2010 | Case-control (2005-2009) | 58±1 | NA | 491 | 167 | 324 | 136 | 355 |
| Pascua et al, 2010 | Case control (2002-2007) | 68.9 | 69.5 | 544 | 174 | 370 | 26 | 518 |
| Fernando et al, 2007 | Case-Control (NA) | 64.2±6.4 | 56.2±1.8 | 233 | 65 | 168 | 78 | 155 |
| Fernando et al, 2013 | Case-control (2007-2010) | 62.4±0.2 | 62.4±1.4 | 318 | 85 | 233 | 190 | 128 |
| Bonderup et al, 2014 | Case-control (2005-2011) | 65.6±3.6 | NA | 7953 | 3884 | 4069 | 5751 | 2202 |

Supplementary Table S1: Characteristics of the studies included in systematic review and meta-analysis.
